# Longitudinal study of respiratory function and symptoms in a non-smoking group of long-term officially-acknowledged victims of pollution-related illness

**DOI:** 10.1186/1471-2458-13-766

**Published:** 2013-08-17

**Authors:** Takako Tanaka, Masaharu Asai, Yorihide Yanagita, Tsuyoshi Nishinakagawa, Naomi Miyamoto, Kenji Kotaki, Yudai Yano, Ryo Kozu, Sumihisa Honda, Hideaki Senjyu

**Affiliations:** 1 Department of Cardiopulmonary Rehabilitation Science, Nagasaki University Graduate School of Biomedical Sciences, 1-7-1 Sakamoto, Nagasaki 852-8520, Japan; 2 Department of Rehabilitation Medicine, Nagasaki University Hospital, 1-7-1 Sakamoto, Nagasaki 852-8501, Japan

**Keywords:** Air pollution, Pollution-related illness, Respiratory function, Respiratory symptoms, Longitudinal study

## Abstract

**Background:**

Air pollution is known to be a leading cause of respiratory symptoms. Many cross-sectional studies reported that air pollution caused respiratory disease in Japanese individuals in the 1960s. Japan has laws regulating air pollution levels and providing compensation for victims of pollution-related respiratory disease. However, long-term changes in respiratory function and symptoms in individuals who were exposed to air pollution in the 1960s have not been well studied. This study aimed to investigate longitudinal respiratory function and symptoms in older, non-smoking, long-term officially-acknowledged victims of pollution-related illness.

**Methods:**

The study included 563 officially-acknowledged victims of pollution-related illness living in Kurashiki, Okayama who were aged ≥ 65 years in 2009. Data were retrospectively collected from yearly respiratory symptom questionnaires and spirometry examinations conducted from 2000 to 2009.

**Results:**

Respiratory function declined significantly from 2000 to 2009 (*p* < 0.01), but the mean annual changes were relatively small. The change in mean vital capacity was −40.5 ml/year in males and −32.7 ml/year in females, and the change in mean forced expiratory volume in 1 second was −27.6 ml/year in males and −23.9 ml/year in females. Dyspnea was the only symptom that worsened significantly from 2000 to 2009 in both sexes (males: *p* < 0.05, females: *p* < 0.01).

**Conclusions:**

Our results suggest that the high concentrations of air pollutants around 1970 resulted in a decrease in respiratory function and an increase in respiratory symptoms in the study population. From 2000 to 2009, the mean annual changes in respiratory function were within the normal range, even though the severity of dyspnea worsened. The changes in respiratory function and symptoms over the study period were probably due to aging. The laws governing air pollution levels and providing compensation for officially-acknowledged victims of pollution-related illness in Japan may be effective for respiratory disease cause by pollution.

## Background

Air pollution is a serious problem throughout the world. Epidemiological studies have reported that air pollution is associated with adverse respiratory effects [1-7] and increased mortality [8-10]. Some areas of Japan experienced high levels of air pollution during the period of rapid economic growth after World War II and many people who lived in these areas complained about respiratory symptoms. In response, the Japanese government implemented air pollution laws. The Basic Law for Environmental Pollution Control was implemented in 1967, and air pollution has decreased since then. The Pollution-Related Health Damage Special Measures Law was implemented in 1969. Japanese citizens who experience health impairment caused by air pollution are certified by prefectural government committees. These officially-acknowledged victims of pollution-related illness qualify for treatment and compensation.

The air in Kurashiki, Okayama had a high sulfur dioxide (SO_2_) concentration in the 1960s because of the high concentration of industrial areas. The number of officially-acknowledged victims of pollution-related illness in Kurashiki peaked at 3,838 in 1988, which was equivalent to 0.9% of the population of the city. An assessment in 2009 revealed that this number had fallen to 1,392. Of the 1,807 individuals (1,118 males, 689 females) who died from 1988 to 2009, the cause of death was available for 501. These 501 individuals were 326 males (65.1%) and 175 females (34.9%) with a mean age at death of 75.3 years (range 15–90 years); the mean age at death was 72.3 years in males and 76.3 years in females. The causes of death were respiratory disease (*n* = 216, 43.1%), malignant neoplasm (*n* = 151, 30.1%), circulatory disease (*n* = 77, 15.4%), and other (*n* = 57, 14.4%). The percentage of people aged ≥ 65 years in Japan increased to 23% in 2009, and a similar pattern of aging occurred in Kurashiki. Of the 1,392 victims of pollution-related illness who were still alive in 2009, 774 (55.6%) were aged ≥ 65 years. The mean age of officially-acknowledged victims of pollution-related illnesses in Kurashiki is increasing, and these individuals are now beginning to develop respiratory illnesses and complications due to aging. These combined health challenges are of major economic and social concern [11].

Many cross-sectional studies have examined the effects of air pollution on respiratory function based on the concentrations or types of pollutants. The Seattle Panel Study [12] reported that the concentration of particulate pollutants was associated with the magnitude of impairment in respiratory function in adults. Almost all studies to date have been cross-sectional, and few longitudinal studies have included measurements of respiratory function. In particular, there is a lack of studies reporting the respiratory symptoms and function of officially-acknowledged victims of pollution-related illness who have received compensation. Furthermore, the effects on respiratory function and symptoms of living in a city that initially had unacceptably high levels of pollution, and later had lower levels of pollution, have not been investigated.

The aim of this study was to conduct a longitudinal assessment of respiratory symptoms and function in older long-term officially-acknowledged victims of pollution-related illness in Japan.

## Methods

### Study design and setting

**Figure 1:**
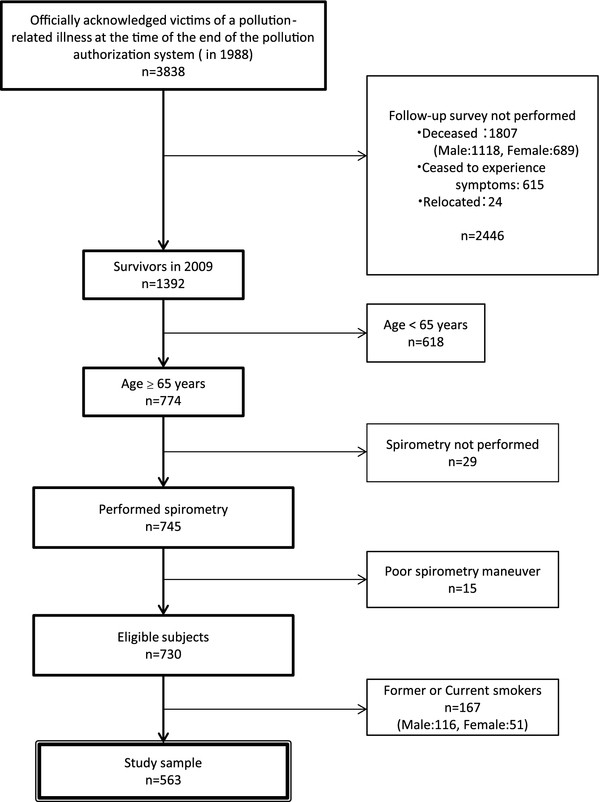
**Flow chart showing selection of the study subjects.** The study included officially-acknowledged victims of pollution-related illness in Kurashiki who were aged 65 years or older in 2009, and for whom the required data for statistical analysis were available.

This study was embedded in a longitudinal study of officially-acknowledged victims of pollution-related illness in Kurashiki from 2000 to 2009. The study protocol was approved by the Ethical Committee of Nagasaki University Graduate School of Biomedical Sciences. The study subjects were drawn from the register of officially-acknowledged victims of pollution-related illness in Kurashiki. Registered victims all met the following conditions as determined by the Public Relief System of Kurashiki City, in accordance with the Pollution-Related Health Damage Special Measures Law (1969) and the Pollution-Related Health Damage Compensation Law (1973): (1) resided or spent time on activities in an area specified as having significant air pollution (Table 1), and (2) were diagnosed with chronic bronchitis, asthma, or emphysema by a doctor. Registered victims were entitled to various forms of compensation including monthly consultation with a doctor, prescriptions for expectorants and bronchodilators, yearly assessment of respiratory symptoms using a detailed questionnaire, and yearly spirometry, in accordance with the Public Nuisance Countermeasures Law. At the time that certification of pollution-related illness ceased in 1988, the population of Kurashiki was 419,203 (204,958 males, 214,245 females), of which 3,838 were officially-acknowledged victims of pollution-related illness (0.9% of the total population). In 2009, the records of these 3,838 victims were reviewed with the authorization of the Kurashiki City Public Office (Figure 1). At that time there were 1,392 registered survivors (634 males and 758 females). The 774 survivors (55.6%) who were aged ≥ 65 years in 2009 (284 males and 490 females) were screened for inclusion in the study. The majority of these victims were diagnosed with chronic bronchitis (*n* = 528, 68.2%), asthma (*n* = 242, 31.3%), or emphysema (*n* = 4, 0.5%) based on their symptom as described in interviews by authorized doctors. Chronic bronchitis was diagnosed if individuals complained of chronic copious sputum production or persistent coughing, asthma was diagnosed if they complained of recurrent episodes of dyspnea and wheezing, and emphysema was diagnosed if the symptoms did not match the criteria for either bronchitis or asthma. Complete spirometry data for the preceding 10 years were available for most victims. To avoid uneven data distribution and selection bias, 44 victims who did not have complete spirometry data for at least 7 of the years from 2000 to 2009 were excluded. A further 167 victims (116 males, 51 females) were excluded because they were former or current smokers. The remaining 563 victims aged ≥ 65 years were included in this study. These victims included 156 males and 407 females who had been certified for 25.9 ± 4.6 years (males: 26.0 ± 4.6 years, females: 25.9 ± 4.6 years). Approximately 60% of these individuals had first been registered as officially- acknowledged victims of pollution- related illness in the 1970s. Date describing spirometry findings and respiratory symptoms were retrospectively collected from the yearly reviews conducted from 2000 to 2009.

**Table 1:** **Time required for certification of pollution**-**related illness**

**Illness**	**a)**	**b)**	**c)**
Chronic bronchitis	24 months	48 months	36 months
Asthma	12 months	30 months	18 months
Emphysema	36 months	66 months	52 months

a) Individual who resided in a designated area prior to 1973.

b) Individual who did not reside in a designated area, but spent at least 8 hours per day in a designated area.

c) Individual who resided in a designated area, then relocated but continued to work in a designated area for at least 8 hours per day.

### Air pollution monitoring

The mean daily concentrations of air pollutants were obtained from instruments installed at 21 points in Kurashiki. Measurement of SO_2_ concentration was started in 1965, and measurement of nitrogen dioxide (NO_2_) concentration was started in 1971.

### Spirometry measurements

Spirometry was performed by trained staff at Mizushima-Kyodo Hospital using an electronic spirometer (FUDAC 70, Fukuda Sangyo Inc., Chiba, Japan). Tests were performed in the sitting position, and were repeated until at least three reproducible forced expiratory curves had been obtained. Forced expiratory volume in 1 second (FEV_1_), forced vital capacity (FVC), and vital capacity (VC) were measured, and the FEV_1_/FVC (%) was calculated. Predicted FEV_1_ was calculated using the equation developed by Berglund et al. [13], predicted FVC was calculated using the equation recommended by the Special Committee of Pulmonary Physiology (Japan Respiratory Society, 1993), and predicted VC was calculated using the equation reported by Baldwin et al. [14].

### Respiratory symptoms

Respiratory symptoms (dyspnea, wheeze, cough, and sputum production) were assessed by physicians during the same season each year, using the respiratory symptoms questionnaire provided by the government of Japan. Each symptom was graded using a standardized 5-point scale, as follows.

#### Dyspnea

1. Too breathless to leave the house, or breathless when dressing or undressing.

2. Breathless after walking about 50 m or after a few minutes on level ground.

3. Breathless when walking on level ground and keeping up with people of the same age, but not breathless when walking at own pace.

4. Breathless when walking up a slight hill or the stairs

5. Breathless only during strenuous exercise.

#### Wheeze

1. Severe episode ≥ 10 days each month during the last year.

2. Severe episode ≥ 5 days each month during the last year, or mild episode ≥ 10 days each month during the last year.

3. Severe episode ≥ 1 day each month during the last year, or mild episode ≥ 5 days each month during the last year.

4. Mild episode ≥ 1 day each month during the last year.

5. No episodes of wheezing.

#### Cough and sputum

1. Daily cough and sputum, with a large amount of sputum or difficulty clearing sputum.

2. Daily cough and sputum, with a moderate amount of sputum or difficulty clearing sputum.

3. Daily cough and sputum, but not troublesome during daily life.

4. Daily cough and sputum for ≤ 3 months each year.

5. No cough or sputum.

### Statistical analysis

The regression coefficients for annual mean changes in FEV_1_, FEV_1_% predicted, FVC, VC, VC % predicted, and FEV_1_/FVC (%) were calculated using simple linear regression analysis. The means and standard deviations were calculated for continuous variables, and a cross-tabulation was constructed for categorical variables. The Kolmogorov-Smirnov test was used to examine the distribution of data. Non-normally distributed data were analyzed using nonparametric tests. Data from the time of certification were compared between males and females using the Mann–Whitney U test and the Kruskal-Wallis test. Data were compared between 2000 and 2009 using the Wilcoxon signed-rank test. Mean annual changes in respiratory function were compared between subjects with and without worsening of dyspnea using the Mann–Whitney U test. All analyses were performed using the PASW software package, version 18. A 2-tailed value of *p* < 0.05 was considered statistically significant.

## Results

### Air pollutants

**Figure 2:**
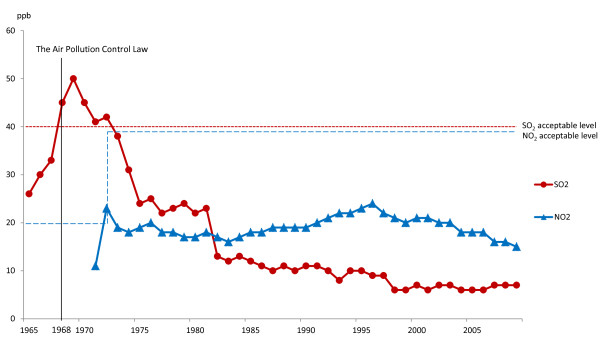
Sulfur dioxide and nitrogen dioxide concentrations from 1965 to 2009 relative to standard values.

Figure 2 shows the annual mean daily levels of SO_2_ and NO_2_ recorded from 1965 to 2009 in Kurashiki. The Air Pollution Control Law was enacted in 1968. The SO_2_ levels were above the acceptable level for all years from 1968 to 1974, and then decreased to below 40 parts per billion (ppb), which is the acceptable level defined by the Air Pollution Control Law. In 1973, the acceptable NO_2_ level was changed by the Air Pollution Control Law from 20 ppb to 40 ppb. The NO_2_ level exceeded the acceptable level only in 1973.

### Patient characteristics at the time of certification

Table 2 shows the characteristics of subjects at the time of certification. Males were significantly younger than females (*p* < 0.05). Males had significantly higher FEV_1_, FVC, and VC than females (*p* < 0.001). Males had significantly lower FEV_1_ % predicted (*p* < 0.001), FVC % predicted (*p* < 0.01), VC % predicted (*p* < 0.01), and FEV_1_/FVC (%) (*p* < 0.05) than females, but these values were all within the normal range. Many subject reported respiratory symptoms: 96.7% of males and 97.8% of females reported dyspnea when walking on level ground and keeping up with people of the same age (grade 1–3 symptoms on the dyspnea scale), 93.5% of males and 96.3% of females reported at least one episode of severe wheezing each month (grade 1–3 symptoms on the wheeze scale), and 64.1% of males and 61.9% of females reported daily cough and sputum production that was troublesome during their daily life (grade 1–2 symptoms on the cough and sputum). These respiratory symptoms were not significantly different between the sexes.

**Table 2:** Patient characteristics at the time of certification

	**Male (n = 156)**	**Female (n = 407)**	***P***
Age (years)	49.6 ± 8.5	51.4 ± 8.9	<.05
Chronic bronchitis	88 (56.4%)	271 (66.6%)	0.395
Asthma	66 (42.3%)	135 (33.2%)	
Emphysema	2 (1.3%)	1 (0.2%)	
FEV_1_ (l)	2.51 ± 0.75	1.93 ± 0.50	<.001
FEV_1_ % predicted	77 ± 21	89 ± 21	<.001
FVC (l)	3.50 ± 0.77	2.59 ± 0.52	<.001
FVC % predicted	91 ± 17	100 ± 16	<.01
VC (l)	3.67 ± 0.74	2.71 ± 0.51	<.001
VC % predicted	102 ± 18	108 ± 17	<.01
FEV_1_/FVC (%)	71.4 ± 14.8	74.5 ± 10.9	<.05
Dyspnea	32 (20.5%)	79 (19.4%)	0.361
	91 (58.3%)	266 (65.4%)	
	28 (17.9%)	53 (13.0%)	
	3 (1.9%)	5 (1.2%)	
	1 (0.6%)	0 (0%)	
Wheeze	47 (30.1%)	161 (39.6%)	<.05
	59 (37.8%)	156 (38.3%)	
	40 (25.6%)	75 (18.4%)	
	8 (5.1%)	13 (3.2%)	
	1 (0.6%)	1 (0.2%)	
Cough and sputum	13 (8.3%)	24 (5.9%)	0.623
	87 (55.8%)	228 (56.0%)	
	49 (31.4%)	144 (35.4%)	
	6 (3.8%)	8 (2.0%)	
	1 (0.6%)	1 (0.2%)	

*FEV*_*1*_ forced expiratory volume in 1 second, *FVC* forced vital capacity, *VC* vital capacity,

*FEV*_1_/*FVC* (%) forced expiratory volume in 1 second.

Values are presented as mean ± standard deviation or number (%).

Male versus female: Mann–Whitney U test, Kruskal Wallis test.

### Respiratory function over time

Table 3 shows spirometry findings in 2000 and 2009 by sex. All measurements of respiratory function declined significantly over time (*p* < 0.01), except for FEV_1_/FVC (%) in both sexes, FEV_1_ % predicted in males, and FVC % predicted in females.

**Table 3:** Comparisons of respiratory function in 2000 and 2009

	**2000**	**2009**	***P***
**a. Male**			
Age (years)	67.0 ± 7.4	75.5 ± 7.3	<.001
FEV_1_ (l)	2.08 ± 0.68	1.82 ± 0.60	<.001
FEV_1_ % predicted	76 ± 23	75 ± 23	0.43
FVC (l)	3.01 ± 0.76	2.67 ± 0.74	<.001
FVC % predicted	90 ± 21	87 ± 22	<.01
VC (l)	3.18 ± 0.73	2.84 ± 0.72	<.001
VC % predicted	98 ± 21	92 ± 21	<.001
FEV_1_/FVC (%)	68.7 ± 13.7	68.4 ± 13.2	0.09
**b. Female**			
Age (years)	68.8 ± 7.8	77.4 ± 7.8	<.001
FEV_1_ (l)	1.59 ± 0.44	1.39 ± 0.46	<.001
FEV_1_ % predicted	93 ± 28	91 ± 23	<.01
FVC (l)	2.23 ± 0.52	1.96 ± 0.56	<.001
FVC % predicted	101 ± 21	100 ± 25	0.62
VC (l)	2.31 ± 0.54	2.04 ± 0.57	<.001
VC % predicted	105 ± 21	99 ± 24	<.001
FEV_1_/FVC (%)	71.3 ± 10.0	71.0 ± 11.3	0.31

*FEV*_*1*_ forced expiratory volume in 1 second, *FVC* forced vital capacity, *VC* vital capacity,

*FEV*_1_/*FVC* (%) forced expiratory volume in 1 second.

Values are presented as mean ± standard deviation.

2000 versus 2009: Wilcoxon signed-rank test.

Table 4 shows comparison of the mean annual changes in spirometry data between results in males and females from 2000 to 2009. Only the mean annual change in VC was significantly greater in males than in females (*p* < 0.05).

**Table 4:** Mean annual changes in the yearly consecutive spirometry data in males and females from 2000 to 2009

	**Male**	**Female**	***P***
FEV_1_ (ml)	−27.6 ± 37.8	−23.9 ± 24.9	0.239
FEV_1_ % predicted	−0.12 ± 1.48	−0.20 ± 1.66	0.221
FVC (ml)	−38.0 ± 47.2	−31.8 ± 34.3	0.104
FVC % predicted	−0.30 ± 1.51	−0.22 ± 1.87	0.354
VC (ml)	−40.6 ± 44.2	−32.7 ± 33.9	<.05
VC % predicted	−0.65 ± 1.39	−0.65 ± 1.59	0.541
FEV_1_/FVC (%)	−0.01 ± 0.77	−0.05 ± 0.75	0.979

*FEV*_*1*_ forced expiratory volume in 1 second, *FVC* forced vital capacity, *VC* vital capacity,

*FEV*_1_/*FVC* (%) forced expiratory volume in 1 second.

Values are presented as mean ± standard deviation.

The regression coefficients for the individual mean annual changes in pulmonary function were calculated using simple linear regression analysis.

Male versus female: Mann–Whitney U test, Kruskal-Wallis test.

### Respiratory symptoms over time

Table 5 shows respiratory symptoms in 2000 and 2009 by sex. There was a significant worsening of dyspnea over this time period (males: *p* < 0.05, females: *p* < 0.01). In females, there was also a significant worsening of wheeze over this time period (*p* < 0.01). There was no significant difference between 2000 and 2009 in cough and sputum production. We examined the relationships between worsening of dyspnea and mean annual changes in spirometry measurements (Table 6). In males, the mean annual changes in spirometry measurements were greater in those with worsening dyspnea than those without worsening dyspnea, but the differences were not significant. In females, however, the mean annual changes in all spirometry measurements except FEV_1_/FVC (%) were significantly greater in those with worsening dyspnea than those without worsening dyspnea (all *p* < 0.05). For the group overall, the mean annual changes in all spirometry measurements, including FEV_1_/FVC (%), were significantly greater in subjects with worsening dyspnea than those without worsening dyspnea (all *p* < 0.05, data not shown).

**Table 5:** Respiratory symptoms in 2000 and 2009

		**2000**		**2009**		***P***
**a. Male**	Score	*n* = 156		*n* = 156		
Dyspnea	1	10	(6.4%)	17	(10.9%)	<.05
	2	91	(58.3%)	92	(59.0%)	
	3	50	(32.1%)	37	(23.7%)	
	4	5	(3.2%)	10	(6.4%)	
	5	0	(0%)	0	(0%)	
Wheeze	1	40	(25.6%)	45	(28.8%)	0.84
	2	60	(38.5%)	53	(34.0%)	
	3	45	(28.8%)	46	(29.5%)	
	4	11	(7.1%)	12	(7.7%)	
	5	0	(0%)	0	(0%)	
Cough and sputum	1	0	(0%)	2	(1.3%)	0.87
	2	77	(49.4%)	75	(48.1%)	
	3	72	(46.2%)	72	(46.2%)	
	4	7	(4.5%)	6	(3.8%)	
	5	0	(0%)	1	(0.6%)	
**b. Female**	Score	*n* = 407		*n* = 406		
Dyspnea	1	35	(8.6%)	41	(10.1%)	<.01
	2	274	(67.3%)	282	(69.5%)	
	3	84	(20.6%)	73	(18.0%)	
	4	13	(3.2%)	10	(2.5%)	
	5	1	(0.2%)	0	(0%)	
Wheeze	1	132	(32.4%)	156	(38.3%)	<.01
	2	159	(39.1%)	137	(33.7%)	
	3	95	(23.3%)	97	(23.8%)	
	4	21	(5.1%)	17	(4.2%)	
	5	0	(0%)	0	(0%)	
Cough and sputum	1	6	(1.5%)	13	(3.2%)	0.97
	2	204	(50.1%)	201	(49.4%)	
	3	183	(45.0%)	182	(44.7%)	
	4	13	(3.2%)	11	(2.7%)	
	5	1	(0.2%)	0	(0%)	

Values are presented as number (%).

2000 versus 2009: Wilcoxon signed-rank test.

**Table 6:** Mean annual changes in the yearly consecutive spirometry data in subjects with and without worsening of dyspnea

	**Without worsening**	**With worsening**	***P***
**a. Male**	*n* = 139	*n*= 17	
FEV_1_ (ml)	−25.6 ± 35.5	−43.2 ± 51.3	0.52
FEV_1_ % predicted	−0.12 ± 1.41	−0.09 ± 2.01	0.63
FVC (ml)	−35.0 ± 43.0	−58.5 ± 64.8	0.29
FVC % predicted	−0.23 ± 1.41	−0.93 ± 2.02	0.32
VC (ml)	−39.1 ± 43.4	−52.9 ± 49.6	0.28
VC % predicted	−0.60 ± 1.37	−1.00 ± 1.60	0.51
FEV_1_/FVC (%)	−0.04 ± 0.72	−0.24 ± 1.10	0.07
**b. Female**	n = 367	n = 40	
FEV_1_ (ml)	−23.1 ± 25.1	−30.9 ± 22.3	<.01
FEV_1_ % predicted	−0.27 ± 1.69	−0.40 ± 1.29	<.001
FVC (ml)	−31.1 ± 35.0	−38.5 ± 26.3	<.05
FVC % predicted	−0.19 ± 1.91	−0.50 ± 1.41	<.05
VC (ml)	−31.4 ± 34.0	−44.6 ± 30.5	<.05
VC % predicted	−0.60 ± 1.59	−1.27 ± 1.45	<.001
FEV_1_/FVC (%)	−0.04 ± 0.73	−0.16 ± 0.90	0.13

*FEV*_*1*_ forced expiratory volume in 1 second, *FVC* forced vital capacity, *VC* vital capacity,

*FEV*_1_/*FVC* (%) forced expiratory volume in 1 second.

Values are presented as mean ± standard deviation.

Without worsening versus with worsening: Mann–Whitney U test.

## Discussion

This paper presents the first longitudinal study of respiratory function and symptoms in officially-acknowledged victims of pollution-related illness who were exposed to air pollution at least 40 years previously, and continued to receive various forms of compensation from the government. Spirometry data at the time of certification, revealed low FEV_1_, FEV_1_ % predicted, and FEV_1_/FVC (%). In addition, a high proportion of victims complained of respiratory symptoms. Many victims were diagnosed with chronic bronchitis or asthma. We consider that the respiratory disease in this group was caused mainly by the high environmental levels of SO_2_ and NO_2_ during 1965 to 1974, because approximately 60% of the victims were first registered during the 1970s. Our results are consistent with those of past reports indicating that high environmental levels of SO_2_ (40–60 ppb) and NO_2_ (17.4 ppb) can affect the bronchi and bronchioles, resulting in respiratory symptoms such as a cough, sputum, wheeze, and breathlessness [1-7,15-17]. Our results show significant differences in respiratory function between males and females at the time of certification, with FEV_1_ % predicted, FVC % predicted, VC % predicted, and FEV_1_/FVC (%) significantly lower in males than in females. These differences are thought to result from the anatomical differences between the sexes. Many studies have shown that lung volumes are smaller in females than in age-matched males [18,19]. Although, FEV_1_ % predicted, FVC % predicted, VC % predicted, and FEV_1_/FVC (%) were significantly lower in males than in females, respiratory symptoms were not significantly different between males and females. This differs from previous reports that females appear to be more significantly affected by air pollution than males [20-22]. It is unknown whether sex affects the defense mechanisms and responses to air pollution in this study. In the current study, most of the males were working or had worked in a factory, whereas about 40% of females were housewives during the 1960s and 1970s [23,24]. The level of exposure therefore differed by sex, with males exposed to more air pollution than females, which would be expected to result in greater impairment of lung function in males [11]. However, we could not definitively determine the reasons for the differences between males and females observed in this study.

Our longitudinal analysis showed a significant decline in respiratory function from 2000 to 2009. The mean annual change in FEV_1_ in this study was −27.6 ml/year in male and −23.9 ml/year in females. The mean annual change in FEV_1_ reported for healthy, non-smoking males and females aged > 65 years is −31 to −22 ml/year [25-27]. The results of the current study were within this range. Furthermore, a previous study [28] reported that patients with chronic obstructive pulmonary disease had a mean annual change in FEV_1_ of 30–80 ml/year, which is 2 or 3 times the mean annual change in of healthy subjects, and higher than the mean annual change of the subjects in this study. The mean annual change in FVC in this study was −33.4 ml/year. The mean annual change in FVC reported for healthy, non-smoking males and females aged > 65 years is −38 to −11 ml/year [25-27,29-31]. The mean annual change in VC in this study was −40.6 ml/year in males and −32.7 ml/year in females. The mean annual change in VC reported for healthy, non-smoking males and females aged > 65 years is −35 to –10 ml/year [25-27,29,31,32]. The mean annual changes in FVC and VC in this study were therefore within the previously reported ranges for healthy, non-smoking males and females aged > 65 years. We considered that aging is the main cause of the decline in respiratory function observed from 2000 to 2009, with almost no additional effect cause by exposure to air pollutions. There are several possible reasons for the preservation of respiratory function in our study population. The level of air pollution in Kurashiki exceeded environmental standards until 1973, after which it declined as a result of the establishment of environmental standards and the introduction of antipollution laws. The NO_2_ level did not exceed the environmental standard from 2000 to 2009, and continued to decrease during this time. The SO_2_ level also did not exceed the environmental standard from 2000 to 2009. Downs et al. [33] reported that a reduction in air pollution may slow the annual rate of decline of respiratory function in adults. Another study showed that pollutant related delay in lung development in children can be attenuated if the children move to cleaner geographic areas [34]. In Switzerland, residence in more polluted areas has been associated with reduced respiratory function in adults [35]. We therefore consider that the study population may not have had a significant pollution- related decline in respiratory function from 2000 to 2009 because the concentrations of air pollutants continued to decrease. Another possible explanation is that medical treatment may have prevented deterioration of symptoms in officially-acknowledged victims of pollution-related illness. Reduced decline in respiratory function has been reported following treatment with bronchodilators such as inhaled corticosteroids, long-acting bronchial anticholinergic agents, and long-acting β2 agonists [30,36-38]. It is possible that non-smoking individuals with pollution-related illness also sought medical treatment at other healthcare institutions, because continuous medical care is guaranteed by the national government. However, this study did not collect detailed data regarding treatment regimen, and we were therefore unable to evaluate the effects of treatment on respiratory function.

Dyspnea worsened significantly in both sexes during from 200 to 2009. The mean annual changes in respiratory function measurements were greater in subjects with worsening dyspnea than in those whose symptoms remained unchanged. However, the mean annual changes in respiratory function measurements in subjects with worsening dyspnea were within the range reported for healthy, non-smoking males and females aged > 65 years. We therefore think that these changes were due to aging. Previous studies [39,40] reported that exacerbation of dyspnea is associated with age-related decline in respiratory function in normal elderly people. In this study, the mean annual changes in respiratory function measurements were greater in those with worsening dyspnea than those without worsening dyspnea in females but not in males. A previous study showed a stronger association between exposure to air pollution and decline in respiratory function in young females than young males [20]. Several studies have also shown that females are more likely to notice a worsening of respiratory function than males [41-44]. This may be because males have a greater tolerance to change in respiratory function [44] In addition, dyspnea which is the subjective perception of respiratory discomfort, is a result of complex and multifocal mechanisms [45]. These include abnormalities in the respiratory control system, neurochemical receptors, ventilation, respiratory muscles, gas exchange, and so on [45]. Therefore, we were not able to determine the reasons for the different relationships between worsening dyspnea and mean annual changes in spirometry findings males and females in this study. Appropriate medical treatment may have prevented the worsening of wheezing (in males) and cough and sputum (in both sexes), but the effects of medical treatment on these symptoms could not be determined in the current study.

In our population of officially-acknowledged victims of pollution-related illness who were living in an area where the level of air pollutants did not exceed the environmental standards and were receiving compensation set by the Air Pollution Control Law and the revised Public Nuisance Countermeasures Law of Japan, treatment measures were considered to be effective, and decreases in respiratory function over time were mild. However, dyspnea worsened significantly in both sexes from 2000 to 2009, and further intervention for dyspnea is required.

This study has some limitations. First, we evaluated only non-smoking officially-acknowledged victims of pollution-related illness, and did not include a control group of subjects who lived in the same area. Second, our study population included more females than males. This may be partly explained by a higher death rate in males, as 65% of the deceased individuals for whom records were available were male. The population of Kurashiki also had a higher proportion of females than males, and more males than females were excluded because they were smokers. Third, spirometry testing was conducted during various seasons. Fourth, differences in social background, employment and lifestyle, which could have resulted in differences in exposure to environmental pollutants, and differences in treatment were not taken into account.

## Conclusion

The results of this study suggest that the high concentrations of air pollutants during the 1970s affected respiratory function. However, the mean annual changes in respiratory function in officially-acknowledged victims of pollution-related illness were within the range of healthy, non-smoking males and females aged > 65 years, even though the severity of dyspnea worsened over time. These results suggest that the changes were limited to the effects of aging. The reduction air pollution levels and the laws regarding pollution-related compensation and treatment in Japan may therefore be effective for reducing respiratory disease cause by pollution.

## Abbreviations

FEV1: Forced expiratory volume in 1 second; FVC: Forced vital capacity; NO2: Nitrogen dioxide; SO2: Sulfur dioxide; VC: Vital capacity.

## Competing interests

The authors declare that they have no competing interests.

## Authors’ contributions

HS was the principal investigator, contributed to the design of the study, handled funding and supervision, and made critical revisions to the manuscript for important intellectual content. TT designed the study, collected the data, analyzed and interpreted the data, and prepared the manuscript. SH interpreted the data, analyzed the data, handled supervision, and made critical revisions to the manuscript for important intellectual content. RK made critical revisions to the manuscript for important intellectual content. MA collected d and interpreted the data, drafted the manuscript and made critical revisions to the manuscript for important intellectual content. YY, TN, NM, KK, and YY collected, analyzed, and interpreted the data. All authors read and approved the final manuscript.

## Pre-publication history

The pre-publication history for this paper can be accessed here:

http://www.biomedcentral.com/1471-2458/13/766/prepub
